# Autoimmune progesterone dermatitis in a patient with endometriosis: case report and review of the literature

**DOI:** 10.1186/1476-7961-2-10

**Published:** 2004-08-02

**Authors:** Alan P Baptist, James L Baldwin

**Affiliations:** 1 Division of Allergy/Immunology, Department of Internal Medicine, University of Michigan, 3918 Taubman Center, #0380, 1500 E Medical Center Drive, Ann Arbor, MI 48109-0380, USA

## Abstract

Autoimmune progesterone dermatitis (APD) is a condition in which the menstrual cycle is associated with a number of skin findings such as urticaria, eczema, angioedema, and others. In affected women, it occurs 3–10 days prior to the onset of menstrual flow, and resolves 2 days into menses. Women with irregular menses may not have this clear correlation, and therefore may be missed. We present a case of APD in a woman with irregular menses and urticaria/angioedema for over 20 years, who had not been diagnosed or correctly treated due to the variable timing of skin manifestations and menses. In addition, we review the medical literature in regards to clinical features, pathogenesis, diagnosis, and treatment options.

## Introduction

While many women complain of worsening acne and water retention during their menstrual cycle, there exist a small number in whom the menstrual cycle is associated with a variety of other skin manifestations such as urticaria, eczema, folliculitis, and angioedema. This condition is known as autoimmune progesterone dermatitis (APD) due to the fact that progesterone is most frequently identified as the etiologic agent. In women with irregular menses, the diagnosis may remain elusive for years. We present a case of APD, and review the current literature in regards to clinical features, pathogenesis, diagnosis, and treatment options.

## Case

A 33y/o woman with a history of endometriosis presented with complaints of chronic urticaria. The patient noted that the urticaria began at the age of 12, and did not seem to have any obvious trigger. Each individual lesion would last from 12–24 hours, and the entire episode would last 5–10 days. Lesions would usually start on the chest and then spread over the entire body. She had seen multiple physicians, including allergists and dermatologists, and had been treated with a variety of medications including certirizine, desloratadine, hydroxyzine, montelukast, ranitidine, and diphenhydramine without relief. Prednisone at high doses would provide temporary relief, and she had required multiple courses of prednisone over the past 20 years. In addition, she complained of occasional angioedema, usually at the same time as the hives but occasionally occurring when hives were not present. The patient also had acne that had been very difficult to control since her teenage years, and she noted that the acne would also respond to prednisone.

Multiple lab tests over the years had been unremarkable. These included SSA/SSB, anti-Smith, ACE level, C3/C4, hepatitis B, ANA, anti double-stranded DNA, immunoglobulins, SPEP, C1 esterase inhibitor level and function, chemistry panel, liver tests, TSH, T4, thyroid antibodies, rheumatoid factor, ESR, and CBC. Skin biopsy of a lesion had been read as "chronic urticaria".

Upon further questioning, it was learned that due to the patient's endometriosis, she had very irregular menstrual cycles in terms of length and timing. It was determined that the hives and/or angioedema would begin approximately 4 days prior to the onset of menses, and would last about 2 days into menses. The symptoms would not occur with every episode of menses. The patient's acne would often occur on her face during the urticarial episodes. Of note, the patient had 2 children, and during each pregnancy her hives, acne, and angioedema had been markedly improved. Because of her endometriosis, she had been started on Depo-Provera (medroxyprogesterone acetate) in her twenties. After 1 injection, she developed severe hives that lasted over 2 months and required multiple courses of prednisone. Due to the urticaria, Depo-Provera was discontinued after one injection. As the patient complained of acne, Ortho Tri-Cyclen (norgestimate/ethinyl estradiol) was initiated by her dermatologist. This treatment modality did not have any effect on the urticaria, angioedema, or acne.

The patient was evaluated in our clinic. Physical examination was essentially normal, and no hives were noted. Allergy skin testing was performed with progesterone 50 mg/mL in normal saline. Prick test was normal, but a full strength intradermal test revealed a 7 mm wheal with erythema. The histamine control showed a 9 mm wheal with erythema, and saline control was negative for wheal and erythema. Two healthy controls also underwent intradermal testing to exclude irritant reaction, and were found to be negative

Based on the above results, the patient was diagnosed with autoimmune progesterone dermatitis. The patient was started on a GnRH agonist (nafarelin acetate nasal spray, 200 mcg twice a day). Within one month, she noted dramatic improvement in her urticaria and angioedema. Acne was still occasionally present, but much improved. She did complain of mild hot flashes, but felt these were tolerable.

## Discussion

In a small group of women, the menstrual cycle has been associated with a spectrum of dermatologic diseases including eczema, erythema multiforme, stomatitis, papulopustular lesions, folliculitis, angioedema, urticaria, and others (Table 1) [1-8]. As progesterone sensitivity has been the most commonly identified cause, dermatologic diseases associated with the menstrual cycle have been labeled autoimmune progesterone dermatitis (APD) [4]. The first documented case of APD was in 1921, in which a patient's premenstrual serum caused acute urticarial lesions. In addition, it was shown that the patient's premenstrual serum could be used to desensitize and improve her symptoms [9]. Since 1921, approximately 50 cases of APD have been published in the medical literature.

**Table 1:** Dermatologic manifestations of autoimmune progesterone dermatitis

- Urticaria
- Angioedema
- Eczema
- Erythema multiforme
- Stomatitis
- Folliculitis
- Papulopustular/papulovesicular lesions
- Stephens-Johnson syndrome
- Vesiculobullous reactions
- Dermatitis herpetiformis-like rash
- Mucosal lesions

### Clinical Features

The clinical symptoms of APD (eczema, urticaria, angioedema, etc.) usually begin 3–10 days prior to the onset of menstrual flow, and end 1–2 days into menses. Severity of symptoms can vary from nearly undetectable to anaphylactic in nature, and symptoms can be progressive [10,11]. There are no specific histological features on biopsy in APD [12]. The age of onset is variable, with the earliest age reported at menarche [13]. Some studies have noted that a majority of patients had taken an oral contraceptive (OCP) prior to the onset of APD [14], but multiple cases exist in which women have never been exposed to exogenous progesterone [15-17].

The symptoms of APD correlate with progesterone levels during the luteal phase of the menstrual cycle. Progesterone begins to rise 14 days prior to the onset of menses, peaks 7 days prior to menses, and returns to a low baseline level 1–2 days after menses begins. In studies where an etiologic agent has been sought, progesterone has been found most frequently. However, estrogen, prostacyclin, and gonadotropin levels have correlated with symptoms in some cases [18-21].

Symptoms may first appear, improve, or worsen during pregnancy and the peripartum period [2,22-24]. In addition, APD during pregnancy has been associated with spontaneous abortions [2,25]. Pregnancy is associated with an increase of maternal progesterone levels, which may explain the initiation or worsening of symptoms. In regards to an improvement of symptoms during pregnancy, a number of theories have emerged. Explanations include a slow rise of progesterone during pregnancy that acts as a method of desensitization, a decrease in maternal immune response during pregnancy, or an increased production of anti-inflammatory glucocorticoids [13,25,26].

### Pathogenesis

The exact pathogenesis of APD is unknown. If exogenous progesterones (i.e. OCPs) are initially used, it is conceivable that uptake by antigen presenting cells and presentation to T_H_2 cells could result in subsequent IgE synthesis; however this mechanism would not explain the pathogenesis in patients such as ours who have the onset of APD prior to exogenous progesterone exposure. Some authors have suggested that hydrocortisone or 17-α-hydroxyprogesterone have cross-sensitivity with progesterone and may cause initial sensitization, but this has not been observed in all studies [27,28].

To further delineate the pathogenesis, antibodies against progesterone have been investigated. Using immunofluorescent techniques and basophil degranulation tests, studies have found that such antibodies do exist in certain patients with APD [1,13,29]. However, negative results looking for antibodies have also been reported [24]. In addition, skin test results with progesterone have shown immediate reactions (within 30 minutes), delayed reactions (24–48 hours later), and reactions with features of both immediate and delayed features [13,14,30,31]. This presumably indicates both type I and type IV hypersensitivity reactions. Progesterone has also been reported to have a direct histamine releasing effect on mast cells, yet very little research has been done to support this hypothesis [32]. Additionally, one study found an in vitro increase of an interferon-γ release assay, possibly implying a role for T_H_1-type cytokines in APD [33].

Eosinophils may also be involved in the pathogenesis of APD. Eosinophilia has been correlated with cutaneous symptoms in some cases, and studies have found a decrease in total eosinophil count after therapy [13,29,34]. Whether increased eosinophils are a response to cytokines from lymphocytes or play a primary mechanistic role in APD remains to be determined.

### Diagnosis

The diagnosis of APD requires an appropriate clinical history accompanied by an intradermal injection test with progesterone. An aqueous suspension or aqueous alcohol solution of progesterone is the preferable vehicle of testing as progesterone in oil can cause an irritant reaction [35], though many published case reports have used progesterone in oil for testing. Various authors have advocated different amounts of progesterone or medroxyprogesterone to be used for testing [12,33,36]. As had been done in some prior studies, the patient presented here was tested with progesterone in aqueous solution at a concentration of 50 mg/mL.

As mentioned above, APD may be due to an immediate or delayed hypersensitivity reaction. Therefore, intradermal testing may not become positive until 24–48 hours later [14,24]. In addition, some authors have advocated patch testing with progesterone to further evaluate for a hypersensitivity reaction [33]. Of note, intradermal testing has been negative in some patients with typical clinical symptoms of APD and who improved after APD treatment [2,3,24].

Some authors have recommended further tests to evaluate the immunologic evidence in APD. These include circulating antibodies to progesterone, basophil granulation tests, direct and indirect immunofluorescence to luteinizing cells of the corpus luteum, in vitro interferon-γ release, and circulating antibodies to 17-α-hydroxyprogesterone [1,7,13,29,33,36]. However, most case reports in the medical literature do not routinely check for serologic evidence of APD, and when checked these markers have not always been found to be reliable. This is most likely due to the fact that, as mentioned above, the pathogenesis of APD is incompletely understood.

### Treatment

Autoimmune progesterone dermatitis is usually resistant to conventional therapy such as antihistamines. The use of systemic glucocorticoids, usually in high doses, has been reported to control the cutaneous lesions of APD is some studies, but not in others [3,10,37]. Early reports of APD describe attempts of progesterone desensitization, and some authors even attempted injections derived from the corpus luteum [18,24,38]. However, results were usually temporary, and such methods of treatment have now fallen out of favor.

Current therapeutic modalities often attempt to inhibit the secretion of endogenous progesterone by the suppression of ovulation. Table 2 lists some of the pharmacologic strategies used in APD. Oral contraceptives (OCPs) are often tried as initial therapy, but have had limited success, possibly due to the fact that virtually all OCPs have a progesterone component. Conjugated estrogens have also been used in the treatment of APD. These did show improvement in many of the patients, but often required high doses [2,16,22]. However, due to the increased risk of endometrial carcinoma with unopposed conjugated estrogens, this treatment is not commonly used today [39].

**Table 2:** Treatment options used in autoimmune progesterone dermatitis

**Treatment Option**	**Advantages**	**Disadvantages**
Oral Contraceptives (OCPs)	- Usually tried as initial therapy	- Limited success due to the progesterone component of OCPs
	- Fewer side effects than other most other therapies	
Antihistamines	- Well tolerated, few side effects	- Rarely effective as monotherapy
		- Does not address underlying mechanism
Conjugated Estrogens	- Avoids progesterone component of OCPs	- Increased risk of endometrial cancer, not commonly used today
		- Often require high doses
Glucocorticoids	- Able to suppress multiple components of the immune system	- Usually not effective alone
	- Can be combined with other therapies	- Often require high doses
GnRH Agonists	- Often used if OCPs and glucocorticoids are not effective	- Can cause symptoms of estrogen deficiency (hot flashes, decreased bone mineral density)
Alkaylated Steroids	- Can be combined with low dose steroids	- Can cause symptoms of excess androgens (facial hair, hepatic dysfunction, mood disorders)
	- Interferes with gonadal hormone receptors	
Tamoxifen	- Has been used successfully in patients unresponsive to conjugated estrogen	- Can cause symptoms of estrogen deficiency
		- Increased risk of venous thrombosis and cataract formation
Bilateral oopherectomy	- Definitive treatment, used if medical options unsuccessful	- Surgical procedure, associated morbidity
		- Symptoms of estrogen deficiency

Various other therapy modalities are currently used in APD, and there is no clear treatment of choice. GnRH agonists, such as buserelin and triptorelin, have been used to induce remission of symptoms by causing ovarian suppression [7,11,15]. However, side effects include symptoms of estrogen deficiency (hot flashes, vaginal dryness, decreased bone mineral density), and estrogen supplementation may be needed [40]. Alkaylated steroids such as stanozol have been used to successfully suppress ovulation, sometimes in combination with chronic low doses of corticosteroids [37]. Side effects of alkaylated steroids include abnormal facial or body hair growth, hepatic dysfunction, and mood disorders, any of which may limit their use. To decrease the risk of side effects, some authors have recommended using the alkaylated steroid only in the perimenstrual period [37]. Another therapeutic option used in APD has been the antiestrogen tamoxifen, which also can suppress ovulation [3,5]. As with GnRH agonists, patients on tamoxifen may experience symptoms of estrogen deficiency. In addition, tamoxifen has been associated with an increased risk of venous thrombosis and cataract formation. In some patients with unremitting symptoms of APD, bilateral oopherectomy has been required [10,15,24]. While this definitive treatment has been successful in controlling symptoms, today it is rarely used before all medical options have been exhausted.

## Conclusion

Autoimmune progesterone dermatitis is a condition seen in a small number of women who present with eczema, erythema multiforme, stomatitis, papulopustular lesions, folliculitis, angioedema, urticaria, and other skin manifestations in relation to the menstrual cycle. It is usually seen 3–10 days prior to the onset of menstrual flow, but may be difficult to recognize in women with irregular menses. The exact pathogenesis is unknown, and is thought to involve a hypersensitivity reaction to progesterone. The diagnosis of APD is made by an appropriate clinical history accompanied by an intradermal injection test with progesterone. Current treatment modalities often attempt to inhibit the secretion of endogenous progesterone, but may be unsuccessful. More research is needed into the pathogenesis of APD to most appropriately care for these patients.
